# Changes in psychiatric diagnoses and mental health care needs after bariatric surgery

**DOI:** 10.1192/j.eurpsy.2026.11047

**Published:** 2026-07-31

**Authors:** M. M. Munoz, A. S. García, C. F. Pato, M. B. Pomar

**Affiliations:** 1 Psychiatry; 2 Endocrinology And Nutrition, Complejo Asistencial Universitario De León, León, Spain

## Abstract

**Introduction:**

Bariatric surgery effectively treats morbid obesity, but its impact on mental health is still debated. Psychiatric comorbidities are common, and surgery may affect—or be affected by—these conditions. This section examines the prevalence, evolution, and treatment of psychiatric disorders in bariatric patients, focusing on diagnostic changes, psychopharmacological management, and psychosocial outcomes such as employment.

**Objectives:**

To assess whether bariatric surgery has a positive effect on psychiatric conditions.

To assess whether patients regain occupational functioning.

To determine whether patients require less psychiatric medication after bariatric surgery.

**Methods:**

This retrospective study of 126 bariatric surgery patients evaluated changes in psychiatric diagnoses, treatments, and employment status before and after surgery. Statistical analyses, including the McNemar test, and visualizations of diagnostic proportions were performed using the AI platform Julius.ai

**Results:**

The sample included 126 patients (90 women, 71.4%; 36 men, 28.6%) with a mean age of 51.96 years (SD = 9.72). Preoperatively, 42.06% had a psychiatric diagnosis, compared to 46.03% postoperatively. Psychiatric diagnoses remained unchanged in 78 cases; 17 new diagnoses appeared, 11 resolved, and 20 changed category. Figure 1 shows these changes. None of the differences were statistically significant (McNemar test, p > 0.05).

A total of 46 patients (36.5%) required psychiatric or psychological support before, during, or after surgery. Psychiatric inpatient admission and suicide attempts occurred in 2.38% of patients each.

Before surgery, 45.24% did not require psychiatric treatment; others used antidepressants (4.49%), anxiolytics (14.29%), combinations of psychotropics excluding antipsychotics (30.95%), or including antipsychotics (3.97%). Postoperatively, no patients used only antidepressants; 31.75% used combinations with antidepressants and anxiolytics or hypnotics, 9.52% used antidepressants with antipsychotics, and 10.32% used anxiolytics or hypnotics alone. The McNemar test found no significant differences in medication class use, indicating bariatric surgery did not reduce psychiatric pharmacological treatment. Figure 2 shows treatment changes.

Among 61 patients with employment data, 21 were unemployed or on disability. Psychiatric medical leave was recorded in 9 patients before surgery and 13 after.

**Image 1:**
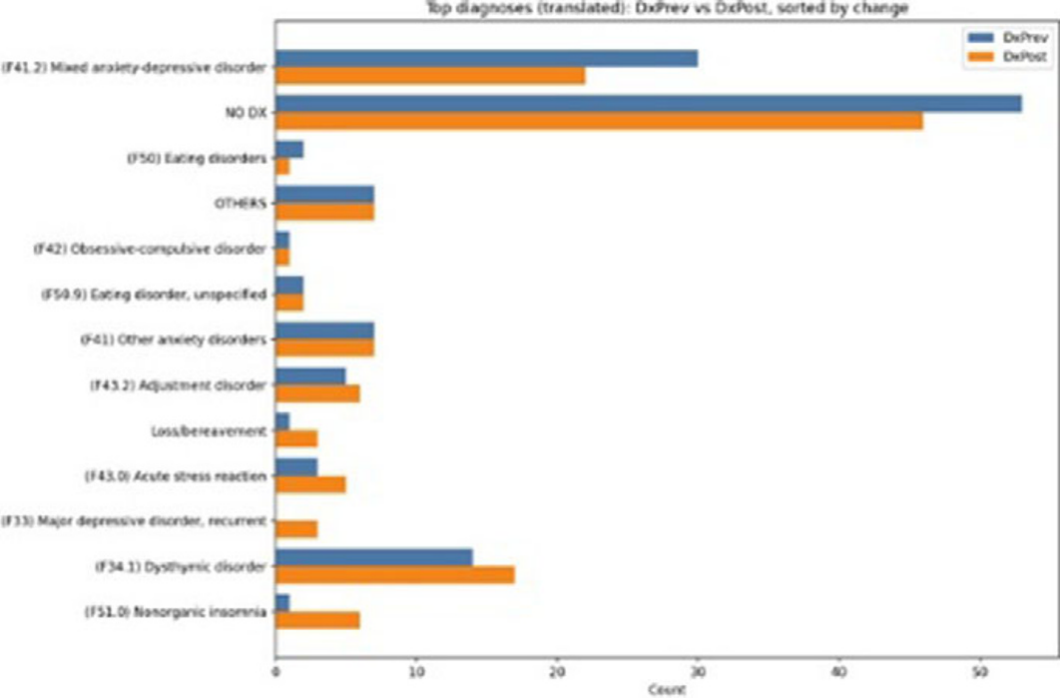
Image 1: Long description.

**Image 2:**
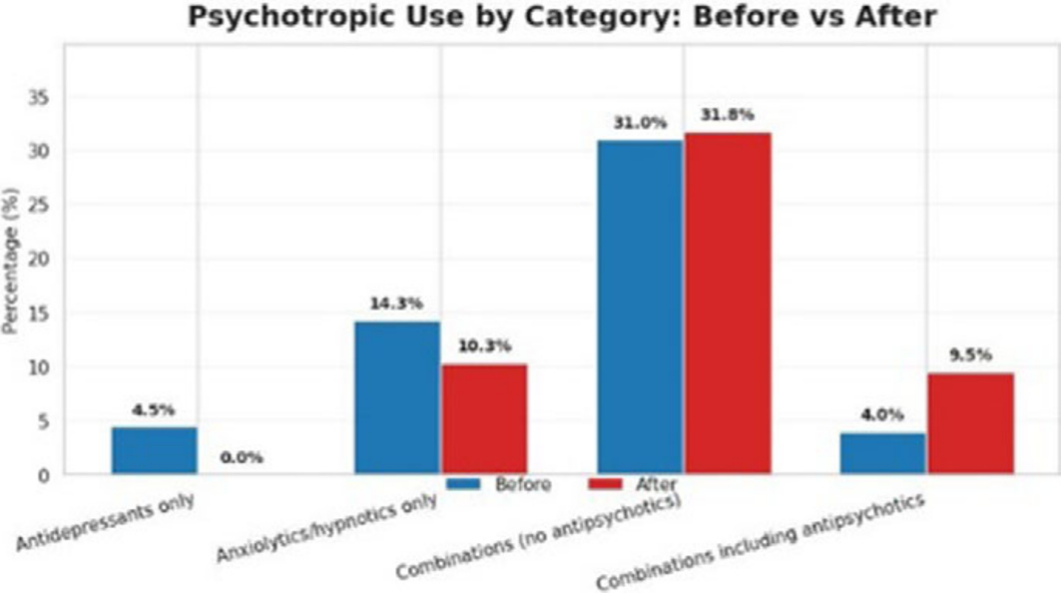
Image 2: Long description.

**Conclusions:**

Improvements in self-esteem, self-care, and self-perception after surgical weight loss were expected to reduce psychiatric pathology. However, bariatric surgery did not significantly change psychiatric diagnoses or medication use. Despite physical benefits, psychiatric conditions—especially anxiety disorders—often persisted or required more treatment, underscoring the need to address mental health. Future studies will explore the link between diagnostic changes and weight loss.

**Disclosure of Interest:**

None Declared

## Long descriptions

### Image 1: Long description

A horizontal bar chart titled Top diagnoses (translated) D x Prev vs D x Post, sorted by change. The x-axis is labeled Count and ranges from 0 to 50. The y-axis lists 13 diagnostic categories. Each category has two bars: a blue bar for D x Prev and an orange bar for D x Post.

* (F 4 1.2) Mixed anxiety-depressive disorder: Blue bar at 30, orange bar at 22.

* N O D X: Blue bar at 53, orange bar at 46.

* (F 5 0) Eating disorders: Blue bar at 2, orange bar at 1.

* O T H E R S: Both bars equal at 7.

* (F 4 2) Obsessive-compulsive disorder: Blue bar at 1, orange bar at 0.5.

* (F 5 0.9) Eating disorder, unspecified: Both bars equal at 2.

* (F 4 1) Other anxiety disorders: Both bars equal at 7.

* (F 4 3.2) Adjustment disorder: Blue bar at 5, orange bar at 6.

* Loss/bereavement: Blue bar at 1, orange bar at 3.

* (F 4 3.0) Acute stress reaction: Blue bar at 3, orange bar at 5.

* (F 3 3) Major depressive disorder, recurrent: Blue bar at 0, orange bar at 3.

* (F 3 4.1) Dysthymic disorder: Blue bar at 14, orange bar at 17.

* (F 5 1.0) Nonorganic insomnia: Blue bar at 1, orange bar at 6.

Navigate back to Image 1:.

### Image 2: Long description

The y-axis is labeled Percentage (%) and ranges from 0 to 35 in increments of 5. The x-axis lists four medication categories. Each category has a blue bar for Before and a red bar for After.

* Antidepressants only: Before is 4.5% and After is 0.0%.

* Anxiolytics/hypnotics only: Before is 14.3% and After is 10.3%.

* Combinations (no antipsychotics): Before is 31.0% and After is 31.8%.

* Combinations including antipsychotics: Before is 4.0% and After is 9.5%.

The data shows a decrease in single-drug use and an increase in complex combinations including antipsychotics.

Navigate back to Image 2:.

